# Air-Pollution Control in an Emergent Market: Does It Work? Evidence from Romania

**DOI:** 10.3390/ijerph17082656

**Published:** 2020-04-13

**Authors:** Ionica Oncioiu, Tatiana Dănescu, Maria-Alexandra Popa

**Affiliations:** 1 Faculty of Finance–Banking, Accountancy and Business Administration, Titu Maiorescu University, 040051 Bucharest, Romania; 2 Faculty of Economics and Law, George Emil Palade University of Medicine, Pharmacy, Science and Technology of Targu-Mures, 540139 Targu Mures, Romania; tatiana_danescu@yahoo.com (T.D.); office.article.igi@gmail.com (M.-A.P.)

**Keywords:** air pollution, sustainable development, green economy, environmental aspects, environmental law, environmental-impact assessment

## Abstract

Economic development in a national and international context must be based on a sustainability strategy established on the systemic interaction between the economic, sociocultural, and ecological environments. Today, the world is confronted by many challenges related to climate change and natural-resource flows, including waste streams resulting from economic activity. The need for national and European environmental standards and the work of an environment monitoring authority to reduce air pollution are highlighted by economic and industrial activities. Thus, our research focused on determining if emissions of sulfur dioxide (SO_2_), nitrogen (NO_2_), and particulate matter 10 (PM_10_) are influenced by planned and unplanned inspections made by competent authorities from Romania. We built a regression model that estimates the influence of economic measures imposed by the authorities on reducing industrial air pollution. Preliminary results showed that the number of inspections negatively influences air pollution, indicating that national and local authorities in Romania are striving to maintain air quality and are conducting more inspections when air pollution is high.

## 1. Introduction

Environmental pollution is one of the most debated problems of the 21st century, constituting a duty for all economic agents; public institutions; and, last but not least, citizens to protect the environment and, implicitly, the health of the population [1,2,3]. The industrial development that the world experienced in the 1980s has left a major imprint on the environment [4,5,6].

In recent decades, measures have been discussed and implemented to combat environmental pollution [7,8,9]. Thus, a global need for environmental-protection regulations is recognized. In this regard, international organizations have developed standards regarding economic development that does not significantly affect the environment [9,10].

Air pollution is a threat not only to the environment but also to human health [11]. Numerous studies have proven the negative impact of air pollution on human health [2,6,9,12,13,14]. The results of a study by Kunzli et al. [15] showed that, in European countries like Austria, France, and Switzerland, air pollution is the cause of death of more than 40,000 people. As well, previous studies in Southeast Asia demonstrated a significant association between an increase in mortality and Pollutants Standards Index [16,17,18,19,20]. Likewise, trends in low birth weight, neonatal deaths, fetal and infant mortality, spontaneous abortion, and the occurrence of birth defects were found to correspond closely with quantities of outdoor pollution [21,22,23,24,25].

According to the other study, many respiratory illnesses or attacks are attributed to air pollution, including bronchitis or asthma attacks [15,26,27,28]. Moreover, recent studies [1,29,30,31] showed that, within the European Union (EU), major health problems are caused by air pollution with particulate matter (PM) and nitrogen oxides (NO_2_), especially respiratory ones.

In 2015, Kelly and Fussell [32] published a paper presenting issues related to the negative influence of air pollution on population health. They found, among other things, that air pollution with PM produces significant negative effects on the health of the population, even at very low concentrations. 

Studies showed the negative impact of economic development on environmental and, especially, air quality [11,28,29,30,31,32,33,34,35]. Ostro [33] published an econometric study applied to a sample of several countries, showing that sulfur oxide particles and carbon emissions increase with economic development, caused mostly by industries. Another study revealed a positive relationship between the probability of conducting an inspection and the size of atmospheric-pollutant emissions [36].

**Figure 1:**
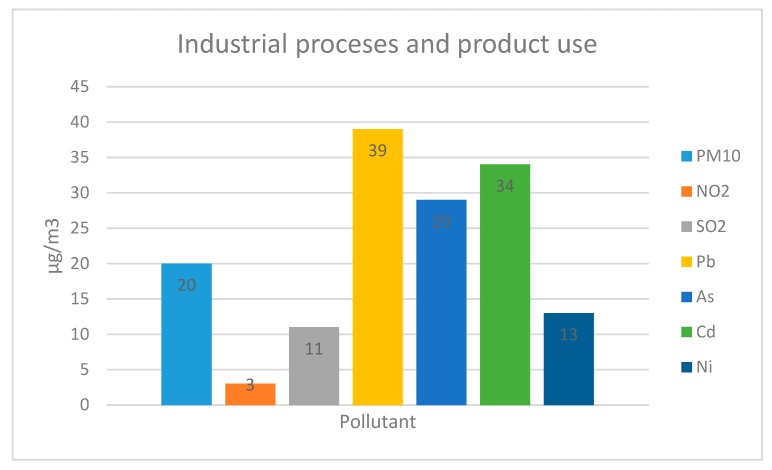
Contribution to EU-28 emissions from the industrial sectors in 2017 of PM_10,_ NO_2_, SO_2,_ As, Cd, Ni, and Pb based on data reported from European Environment Agency (EEA) [38].

In the EU, air pollution due to industrialization produces significant negative effects on human health [37]. According to the report of the European Environment Agency (EEA) on air quality [38], in 2018, the most serious health problems of Europe’s population were caused by PM, NO_2_, and ozone (O_3_) emissions because industry is the third most polluting sector in Europe (Figure 1).

Therefore, the need for the constant monitoring of air pollution and for taking actions on top of monitoring is known to prevent the occurrence of diseases among the population and to protect the environment and ecosystems. Consequently, emissions of polluting factors recorded by companies are closely monitored by the EEA, with preventive and control measures being established. These measures are implemented by national or local authorities.

It was found that a competent authority is the one that can stimulate companies to comply with environmental norms. However, due to specific problems to emerging economies, alignment with imposed international norms tends to be delayed, thus affecting the environment and implicitly the health of the population [5,10,27].

Romania is an emerging market country that uses economic liberalization as the main engine for economic growth. Compared to other EU Member States, Romania is experiencing a high level of pollution from industrial activities, which may make national authorities’ control activities difficult [39,40]. For this reason, through the transposition of the European legislation, industrial stations (among other types of stations), located in key points throughout Romania, register specific air pollutant concentrations that are being considered representative to determine the level of pollution produced by industries.

Given that entities operate in a dynamic and competitive business environment, they always find innovative solutions to avoid compliance with national and/or international norms [41]. The economic performance of entities tends to decrease ecological performance, indicating that efforts made by managers to obtain good economic results could exceed efforts made to protect the environment [5,34,42].

This research analyzes the effectiveness of economic measures applied by the Romanian authorities to reduce industrial air pollution, both in terms of controls carried out by a national authority (the number of carried-out planned and unplanned inspections) and their consequences, represented by the number of imposed fines. The value of the imposed fines, the number of issued warnings, and the number of activity breaks and of the average annual values of the main indicators that compose the air-quality index (mainly influenced by industrial activity), namely, the average annual atmospheric concentration of SO_2_, NO_2_, and PM_10_, were analyzed for a period of thirteen years (2005–2017). As far as we know, no similar study has been conducted in Romania before.

On the other hand, this study contributes to the development of the specialized literature by developing a case study on an emerging economy analyzing the influence of imposed economic measures by the Romanian national control authority on air pollution caused by the industry.

Furthermore, the aim of this study was to identify the influence of economic measures on the level of air pollution, in particular, on the effect of undertaken inspections by the authority in reducing air pollution from industry sources. This study also provides insight into the spillover effects that actual air pollution in Romania might have on public concern about air quality.

The rest of this paper is organized as follows: Section 2 presents the relevant legislative aspects on an emerging EU market. Section 3 discusses the literature review. Section 4 describes the empirical model and the used data in the analyses and develops the hypotheses. The estimated results of the empirical model are reported in Section 5. Section 6 concludes the paper and talks about limitations and directions for future research.

## 2. Relevant Legislative Aspects on an Emerging EU Market

Air pollution is a crucial problem and ongoing issue that has been affecting ecosystems and human health [28,43]. One of the EU’s sustainable-development goals regarding pollution is to reach levels of pollutant emissions that do not constitute a significant risk for the occurrence of health events.

In this respect, each member state of the European Union, by applying European Directive 2004/35/EC, has a delegated authority responsible for checking the compliance of economic agents with national and European pollution norms. Velders et al. [44] suggested that European air-pollution regulations in recent decades have contributed to reducing NO_2_, PM, and SO_2_ emissions.

Regulation on air-pollution control [38] provides information on air-quality assessment on the territory of Romania on the basis of common criteria and methods that were established at the European level (Table 1).

In this sense, over 100 stations annually evaluate the quality of the air throughout the national territory by continuously measuring the concentrations of harmful air pollutants such as sulfur dioxide (SO_2_), nitrogen oxides (NO_2_), ozone (O_3_), carbon monoxide (CO), suspended particulate matter (PM_2.5_ and PM_10_), lead (Pb), and benzene (C_6_H_6_). These stations are strategically dispersed throughout the country, measuring airborne noises from several perspectives, such as industrial, urban, urban background, and rural stations. Nevertheless, there are situations where daily or annual limits exceed the European average. In this regard, the Romanian legislation has designated a national authority, the National Environmental Guard (GNM), to be responsible for controlling environmental pollution.

One of the objectives of national authorities is to improve air quality through the control of industrial pollution. Therefore, the GNM is the Romanian authority designated for the control and evaluation of economic agents regarding compliance with environmental norms. Thus, annually, this authority carries out numerous inspections, both planned and unplanned (for example, when reporting an exceedance of the maximal limit allowed by pollutant emissions), throughout the country. Following controls, if violations of environmental norms are found, the authority applies sanctions to entities responsible for environmental damage in accordance with European Directive 2008/99/EC on environmental protection through criminal law (fully transposed into national law by Law 10/2011), which leaves it to each member state to determine the manner and level of applicable sanctions.

The European Commission reported in 2016 that it was willing to support member states for a better implementation of the imposed European environmental standards. Romania was mentioned in several actions suggested by the Commission in three important areas: the development of the circular economy and the improvement of resource efficiency, waste management, and compliance assurance (http://ec.europa.eu/environment/eir/index_en.htm) [45].

In 2018, Romania and eight other EU Member States convened at the Ministerial Summit on Air Quality held in Brussels to find solutions to the serious problem of air pollution (press release of the European Commission: http://europa.eu/rapid/press-release_IP-18-348_en.htm) [46]. At this summit, the European Commission presented a new action plan to ensure compliance with European air-pollution law by issuing a set of specific measures designed to improve compliance with EU rules. Suggested actions by the European Commission to be undertaken by member states to implement all rules and requirements regarding environmental issues were also included.

## 3. Literature Review

Over time, many researchers have investigated issues related to the compliance of companies with imposed pollution norms as well as the effects of inspections and controls carried out by national authorities [8,13,27,33,36,43,47]. Literature research usually looks at issues of a legal rather than economic nature [2,5,42]. Therefore, in this section, we present studies on the effect of inspections on the degree of compliance with pollution norms.

By analyzing the literature, we found studies that investigated the monitoring of air pollution in terms of regulation [7,9,48,49]. Research from the 1990s showed that traditional national authorities do not cope with pollution caused by industry, so controls that they carry out are not enough to stimulate companies to comply with environmental standards [50,51,52,53]. A study by Dasgupta [46] that analyzed factors that determine how authorities start to control activities showed that a higher effort by authorities to monitor air pollution was found in companies that were predisposed to pollute to a greater level. Telle [54] analyzed a panel dataset that included observations between 1989 and 2001 on emissions and inspections of over 100 Norwegian companies, finding that the probability of conducting inspections decreases the likelihood of regulation violations.

The carried-out inspections can have consequences consisting of sanctions applied to those entities that do not comply with environmental norms. Thus, some researchers say that sanctions granted to companies reduce the rate of infringement [55,56], while other researchers believe that there is a set of components that determine compliance and that the main motivation is related to legal constraint [2,57].

A study by Becker et al. [58] showed that companies’ decisions to reduce environmental pollution seem to be strongly influenced by the size of fines for noncompliance. Similar results were also obtained by Downing and Watson [59], who analyzed the effect of tightening controls performed by United States (U.S.) authorities on companies that pollute excessively on future emissions of air pollutants.

In 2014, Greenstone and Hanna [60] studied the impact of sanctions and inspections carried out in the decision of companies to comply with environmental norms in terms of effects produced by the probability of carrying out inspections and the level of potential fines.

Other authors reported that short-term fluctuations in actual pollution level and concern about pollution were strongly and positively correlated but that long-term annual trends of these two variables were opposite [7,14,61].

At the national level, various studies [62,63,64,65,66] were carried out regarding the level of environmental pollution and its reduction measures but without observing whether this level is influenced by economic measures taken by national authorities. For this reason, as far as we know, the present study is innovative in nature, being the only one that analyzes the national-level impact of economic measures taken by the National Environmental Guard on air quality and the emissions of polluting agents.

## 4. Materials and Methods

To carry out multidisciplinary research, we have combined elements from the fields of economics and environmental protection. In searching for the answer to the research objective, we identified possible variables studied during the 13-year period that had legislative specificities that could be grouped in two time intervals. The used variables, calculation method, and their sources are described in Table 2.

To avoid bias and data deficiencies caused by calculation-result accuracy, 2005 and 2006 were considered important years for the national economy because, in this period in Romania, it was necessary for accession to the European Union to harmonize national with European legislation. The 2007–2017 timeframe represents years in which Romania had joined the European Union.

Considering previous research results, we can start from the idea that the monitoring and control actions of the authorities reduce air pollution. Thus, in achieving the research objective, it was necessary to verify the following economic assumptions:

**Hypothesis 1** **(H1).** *An increase in the number of planned inspections leads to a decrease in air pollution.*

**Hypothesis 2** **(H2).** *An increase in the number of unplanned inspections causes a decrease in emissions of air pollution.*

**Hypothesis 3** **(H3).** *Increasing the share of fines in all inspections causes a decrease in air pollution.*

**Hypothesis 4** **(H4).** *Increasing the weight of warnings in all inspections causes a decrease in air pollution.*

These assumptions are completed by ceteris paribus assumption, which means that our model can contain other variable influences, but these influences remain unchanged during our analysis period.

### 4.1. Dependent Variables

**Figure 2:**
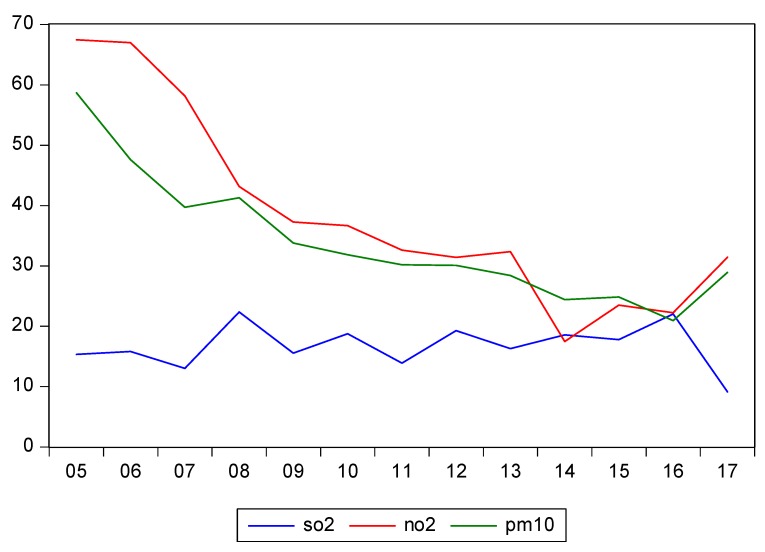
Evolution of dependent variables for 2005–2017 period projection using Eviews based on data collected from European Environment Agency (EEA) reports [38].

The selected dependent variables belong to the environmental-protection field, related to the annual mean of air pollutants concentrations recorded by industrial stations. Air pollution data are collected accessing the interactive map from the website of the European Environmental Protection Agency [38]. For the analyzed period (2005–2017), we extracted data on the minimum and maximum average values recorded by every reporting industrial station for Romania in order to calculate an annual average of air pollution (the arithmetic mean between the minimum average value and the maximum average value recorded by the industrial reporting stations). The industrial stations are strategically placed in key points for recording the concentrations of air pollutants in the environment, such as SO_2_, NO_2_, and PM_10_. The measure unit for dependent variables’ values is µg/m^3^. Figure 2 illustrates the evolution of the SO_2_, NO_2_, and PM_10_ concentrations in the air reported by Romanian industrial stations. We can note a significant decrease of NO_2_ and PM_10_ air concentrations with the accession of Romania to the European Union, starting with 2007.

### 4.2. Independent Variables

The selected independent variables belonged to the economic field, composed of the number of fines granted for noncompliance, the amount of fines (in millions of dollars), the number of cases of activity cessation, the number of issued warnings, the number of verified companies, the total number of inspections, and the number of planned and unplanned inspections out of total carried-out inspections (extracted from the website of the National Environmental Guard [67]).

Planned inspections represent inspections found within the general annual plan of activities of the National Environmental Guard and that cover the whole national territory. Unplanned inspections include conducted inspections for compliance with national air-pollution regulations, authority self-assessments, complaints to verify compliance with previously imposed penalties and measures, and conducted inspections with other regulatory authorities. Total inspections represent the sum of carried-out inspections in a year, both planned and unplanned. Table 3 contains data on independent variables studied in 2005–2017.

### 4.3. Methods

For the data considered at the national level, observations and calculations were made for each of the 13 analyzed years. For the dependent variables, this study aimed at analyzing SO_2_, NO_2_, and PM_10_ air concentrations recorded by industrial stations. For the independent economic variables, it was determined that they were stationary, and a correlation test was performed, finding that only 5 of the 10 variables were not correlated with each other. In this case, we eliminated the intercorrelated variables, with the sample consisting of the number of planned and performed inspections (planned_insp), the number of unplanned and performed inspections (unplanned_insp), total performed inspections (total_insp), amount of fines (fee_val), number of issued warnings from total inspections (warnings_in_total_insp), and number of imposed fines from total inspections (fees_in_total_insp) in relative sizes.

In order to avoid the heteroscedasticity phenomenon, we used the quantitative variables in logarithmic sizes. As independent variables, we used weight values in the case of fines and warnings.

Using the linear-regression model, the influence of independent economic variables on SO_2_, NO_2_, and PM_10_ emissions was analyzed as follows.

Yi = β0 + β1 × Xi + εi(1)

where Y_i_ = annual mean value for each analyzed pollutant (SO_2_, NO_2_, and PM_10_) and X_i_ = independent variable value used for the estimated model.

We chose as working model the ordinary least squares (OLS) model due to the type of data analyzed. The input-output data are represented by logarithmic or relative values, as the case may be. Due to the fact that our quantitative data may produce a relatively constant evolution over time, the linear OLS method can correctly estimate the parameters of the linear function of the analyzed data set. Ceteris paribus assumption is used.

## 5. Results and Discussion

This section reports the results of the empirical analyses. Table 4 contains a summary of the trend of the independent variables included in the study.

Thus, we observed that, during the analyzed period from year to year, there were no significant changes in the number of economic measures imposed by the GNM.

Analyzing the evolution of the number of fines, in 2017, compared to 2005, there was a 156% increase. During the same period, there was a decrease in the number of inspections, both planned and unplanned. Thus, the weight of the number of imposed fines by the GNM in total inspections decreased by 28% in 2017 compared to 2005.

The number of warnings decreased by 42% in 2017 compared to 2005. Thus, the number of issued warnings by the GNM to companies that did not comply with imposed environmental norms in total performed inspections decreased by 41% in 2017 when compared to 2005.

Figure 2 shows that NO_2_ and PM_10_ emissions drastically decreased in the period following Romania’s accession to the EU when compared to the previous period. Thus, starting with 2009, Romania annually reported lower concentrations of NO_2_ and PM_10_ compared to the preaccession period in the EU, which is within the acceptable limits of the European Commission.

Next, in order to be able to estimate the linear model, we established the correlation matrix of the used independent economic variables, as shown in Table 5.

The stationarity of the introduced variables in the estimated models was tested, establishing that the independent variables were stationary.

In the analyzed context, from analysis of the Pearson coefficients, there was a strong connection between unplanned and planned carried-out inspections and the inspection total and between the value of imposed fines and the weight of the warnings in the total performed inspections, for which they are not included in the same econometric model.

Table 6 reflects the obtained results by applying the linear-regression model for each dependent variable (SO_2_, NO_2_, and PM_10_).

Table 6 shows that each calculated coefficient was positive, which means that the dependent variable increased when the independent variable increased. Inspections, both planned and unplanned, were more numerous when SO_2_ air concentration were high. Thus, results showed that the national authority responsible for controlling air pollution conducted inspections when SO_2_ air concentration released into the air were high and air quality was worse. The estimated and found models in Table 6 support this result.

Thus, an increase in planned inspections by 1 caused an increase in SO_2_ air concentration by 0.063, and an increase in unplanned inspections caused an increase in SO_2_ air concentration by 0.072. From an economic point of view, these results showed that national authorities are notified and carried out planned inspections (included in the authorities’ action plan) when SO_2_ air pollution is high. These results were unexpected but similar to those obtained by Telle in 2004, where the likelihood of inspections increased when pollution was also increased.

According to the correlation matrix (see Table 5) between PM_10_ air concentration and NO_2_ air concentration and planned inspections, a strong positive correlation link (0.75 and 0.73, respectively) was established, for which these variables could not be included in the same linear model. However, correlation between them suggests that PM_10_ air concentration and the number of planned inspections depend on each other, influencing them positively. Moreover, a strong positive correlation between PM_10_ and NO_2_ proves to us that these two types of atmospheric emissions are interrelated and mutually determined because these pollutants comes from Diesel exhaust emissions of transportation.

The value of R^2^ in Table 6 indicated that the number of total inspections (either included as such or included as number of planned inspections or number of unplanned inspections) is highly correlated with the SO_2_ air concentrations reported by the industrial stations analyzed. This result is somehow expected due to the fact that authorities must plan their controls based on some pollution background. Although the results are statistically correct *(F*-test shows a *p*-value lower than 0.5), due to the high correlation between dependent and independent variables, we cannot consider these results obtained. 

In the economic context, inspections and the weight of sanctions in total inspections increase when recording high values of SO_2_, NO_2_, and PM_10_. Specifically, the share of fines in the total performed inspections is high when SO_2_ air concentration are high. The same result could be observed in the case of NO_2_ and PM_10_ air concentration. In contrast, the weight of warnings in all inspections only significantly influenced NO_2_ air concentration. In this respect, authorities tend to impose harsher sanctions when the NO_2_ value is high, although it was found that there is no significant influence between the number of performed inspections and the value of these air concentration.

From those presented, national authorities act according to pollution level, carrying out several controls to increase air concentration. Thus, the weight of warnings in total performed inspections by GNM does not significantly influence SO_2_ and PM_10_ air concentration but only NO_2_. However, the test cannot be considered due to the very high value of R^2^, which indicated a high level of correlation between the variables included in the regression.

Following analyses, Table 7 presents the status of the economic hypotheses of the present research.

All four economic hypotheses of the research were rejected, thus observing that, at the national level, GNM efforts to reduce air pollution by carrying out inspections and by granting sanctions are not sufficient. Even so, to support this assertion, further testing and extension of research is needed.

On the basis of the above findings, the government must continue to implement air-pollution control policies and to manage environmental quality to reduce the risks to human health.

## 6. Conclusions

The European Commission is concerned with supporting and advising member states for better and more uniform harmonization of national and European law. In order to align with European legislation on the subject of the present research, Romania must follow actions suggested by the European Commission regarding developing a circular economy; improving the efficiency of resources and waste management; and ensuring compliance.

The research results highlight that national authorities endeavor to conserve the environment by carrying out planned and unplanned inspections within economic agents throughout the country. There was a decrease in inspections and sanctions granted during the analyzed period that could be attributed to the reduction of air pollution as a result of the implementation of European legislation that required compliance with certain thresholds for these polluting-factor emissions. The efforts by authorities to constrain economic agents were insufficient.

Our results show that the industrial air pollution control activities are more rigorous when a high level of pollution is reported. Furthermore, the air pollution with PM_10_ and NO_2_ tend to reduce in post-accession to the EU period while descriptive statistics and Table 4 show us that the number of inspections remained approximately the same in the same period. Thus, we find that authorities’ efforts to constrain economic agents seem to be insufficient to strike down air pollution caused by industrial activities.

The established economic hypotheses at the beginning of the research were not accepted, so the results of the research were not the expected ones but were similar to some studies carried out by other researchers for different countries in Europe [11,13,49,68].

From a practical perspective, this could help Romanian authorities to better understand the degree of public concern about air quality and to better assess current environmental management practices. In terms of scientific significance, this study analyzed the impact on public health related to PM_10_ on the basis of air-pollution research and expands its depth.

In a future study, we intend to carry out more in-depth analysis of the effect of planned and unplanned inspections on air pollutant concentrations and on county air-quality indicators, focusing on counties of which most business was carried out in the industry.

The limits of our research consist of the small number of variables included in the model and the short period analyzed, mainly caused by insufficient available data; the probability of confounding factors that determine the trend of air pollutant concentrations; the changes in the industrial sector (most probably increasing dominance of less polluting sectors, except transportation) over the examination period; or the limitation of how representative the air pollution data are for the whole country.

## Figures and Tables

**Table 1:** Air quality standards for the protection of health.

Pollutant	Averaging Period	Legal Nature and Concentration	Comments
PM_10_	1 day	Limit value: 50 μg/m^3^	Not to be exceeded on more than 35 days per year
	Calendar year	Limit value: 40 μg/m^3^	
NO_2_	1 h	Limit value: 200 μg/m^3^	Not to be exceeded on more than 18 h per year
		Alert threshold: 400 μg/m^3^	To be measured over 3 consecutive hours over 100 km^2^ or an entire zone
	Calendar year	Limit value: 40 μg/m^3^	
SO_2_	1 h	Limit value: 350 μg/m^3^	Not to be exceeded on more than 24 h per year
		Alert threshold: 500 μg/m^3^	To be measured over 3 consecutive hours over 100 km^2^ or an entire zone
	1 day	Limit value: 125 μg/m^3^	Not to be exceeded on more than 3 days per year
Pb	Calendar year	Limit value: 0.5 μg/m^3^	Measured as content in PM_10_
As	Calendar year	Target value: 6 ng/m^3^	Measured as content in PM_10_
Cd	Calendar year	Target value: 5 ng/m^3^	Measured as content in PM_10_
Ni	Calendar year	Target value: 20 ng/m^3^	Measured as content in PM_10_

Source: European Environment Agency (EEA) reports 2019 [38].

**Table 2:** Variable description.

Used Variable	Variable Symbol	Description	Source
		**Dependent Variable**	
NO_2_ concentration in the air	NO_2_	Mean value of annual NO_2_ concentration in the air reported for Romania resulting from industrial activities: For the mean value of annual concentration in the air calculations, we used data found in the European Environmental Agency database, reported by stations placed near industrial activities.	European Environmental Agency reports
SO_2_ concentration in the air	SO_2_	Mean value of annual SO_2_ concentration in the air reported for Romania resulting from industrial activities: For the mean value of annual concentration in the air calculations, we used data found in the European Environmental Agency database, reported by stations placed near industrial activities.	European Environmental Agency reports
PM_10_ concentration in the air	PM_10_	Mean value of annual PM_10_ concentration in the air reported for Romania resulting from industrial activities: For the mean value of annual concentration in the air calculations, we used data found in the European Environmental Agency database, reported by stations placed near industrial activities.	European Environmental Agency reports
		**Independent Variable**	
Planned inspections	Planned_insp	Total of planned inspections carried out by local authorities of the National Environmental Guard throughout Romania for air-pollution control	Annual reports of the National Environmental Guard (GNM)
Unplanned inspections	Unplanned_insp	The total of unplanned inspections carried out by local authorities of the National Environmental Guard throughout Romania for air-pollution control	Annual reports of the National Environmental Guard (GNM)
Total inspections	Total_insp	Total of planned and unplanned inspections carried out by local authorities of the National Environmental Guard throughout Romania for air-pollution control	Annual reports of the National Environmental Guard (GNM)
Fines in total carried-out inspections	Fees_in_total_insp	Total number of fines imposed by local authorities following controls carried out on air pollution	Annual reports of the National Environmental Guard (GNM)
Warnings in total carried-out inspections	Warnings_in_total_insp	Total number of issued warnings by local authorities following air-pollution controls	Annual reports of the National Environmental Guard (GNM)

**Table 3:** Descriptive statistics of the variables included in the study.

Variable	Mean	Median	Standard Deviation	Max	Min
SO_2_ (µg/m^3^)	16.748	16.300	3.638	22.370	9.110
NO_2_ (µg/m^3^)	38.512	32.610	16.247	67.450	17.480
PM_10_ (µg/m^3^)	33.898	30.190	10.482	58.660	20.930
Planned_insp (no)	14,348.46	13,822.00	2386.891	19,018	10,455
Unplanned_insp (no)	24,673.08	23,592.00	5112.006	34,072	19,173
Total_insp (no)	39,021.54	39,645.00	6202.715	47,894	30,876
Fees_in_total_insp (%)	27.009	23.640	8.096	41.060	17.540
Warnings_in_total_insp (%)	5.182	4.890	1.511	9.160	3.200

Number of planned inspections (planned_insp); number of unplanned inspections (unplanned_insp); number of total performed inspections (total_insp); amount of fines (fee_val); number of issued warnings from total inspections (warnings_in_total_insp). Source: authors’ research and projection based on GNM national reports [67] and data collected from European Environment Agency (EEA) reports [38].

**Table 4:** Evolution of analyzed variables.

Period	No. Charges and Fees	No. Warnings	No. Planned Inspections	No. Unplanned Inspections	Fees and Charges of Total Inspections (%)	Warnings of Total Inspections (%)
2006/2005	0.05	−0.14	0.06	0.03	0.16	−0.45
2007/2006	−0.27	0.05	−0.13	−0.05	0.59	0.12
2008/2007	−0.11	0.45	−0.19	−0.06	0.46	−0.19
2009/2008	−0.26	−0.24	−0.02	0.15	−0.16	−0.03
2010/2009	0.81	0.16	0.10	0.33	−0.09	−0.24
2011/2010	0.01	−0.17	−0.04	0.03	0.16	0.01
2012/2011	−0.53	−0.36	0.11	−0.08	−0.15	0.05
2013/2012	0.04	0.10	−0.19	−0.38	0.35	1.83
2014/2013	−0.30	−0.22	−0.16	0.06	0.36	−0.22
2015/2014	0.18	−0.06	0.22	−0.01	−0.24	−0.05
2016/2015	0.06	0.09	−0.02	−0.05	−0.09	0.47
2017/2016	0.14	0.24	0.00	0.01	0.05	−0.09
2017/2005	1.56	0.58	0.87	0.55	0.72	0.59

Source: authors’ own calculations and projections based on GNM national reports [67].

**Table 5:** Correlation matrix for variables included in linear regression.

	Variable	SO_2_ (1)	SO_2_ (2)	SO_2_ (3)	SO_2_ (4)	SO_2_ (5)	NO_2_ (6)	NO_2_ (7)	PM_10_ (8)
**(1)**	total_insp								
**(2)**	planned_insp	0.61							
**(3)**	unplanned_ insp	0.93	0.27						
**(4)**	fee_in_total_ insp	0.65	0.47	0.56					
**(5)**	warnings_in_total_insp	−0.04	0.05	−0.07	0.49				
**(6)**	fee_val	0.29	0.02	0.35	0.59	0.69			
**(7)**	SO_2_	0.03	0.13	−0.01	0.01	0.28	0.26		
**(8)**	NO_2_	0.48	0.75	0.55	0.38	0.42	0.16	−0.26	
**(9)**	PM_10_	0.43	0.73	0.18	0.35	0.56	0.10	−0.18	0.934

Source: Authors’ own calculations and projections.

**Table 6:** Linear-regression estimations.

Variable	SO_2_ (1)	SO_2_ (2)	SO_2_ (3)	SO_2_ (4)	SO_2_ (5)	NO_2_ (6)	NO_2_ (7)	PM_10_ (8)
total_insp			0.095 ** (0.009)					
planned_insp	0.063 * (0.012)			0.036 ** (0.003)				
unplanned_insp		0.072 ** (0.006)		0.061 ** (0.005)				
fees_in_total_insp					0.001 * (0.025)		0.019 * (0.034)	0.026 * (0.047)
warnings_in_total_insp						0.175 ** (0.000)		
F–statistic	0.011	<0.001	<0.001	<0.001	0.017	<0.001	0.037	0.010
R^2^	0.456	0.868	0.999	0.999	0.417	0.967	0.338	0.465

Note: ** significance at 0.01, * significance at 0.05. Values in brackets represent *p*-value. Source: authors’ own calculations and projections.

**Table 7:** Accepted/rejected hypotheses.

	Accepted/Rejected	
Hypotheses	National Level	Observations
H_1_	Rejected	Planned inspections do not cause a decrease in the level of air pollution.
H_2_	Rejected	Unplanned inspections do not cause a decrease in the level of air pollution.
H_3_	Rejected	The increase of the weight of the fines in the total inspections carried out does not lead to a reduction of the pollutant air concentration
H_4_	Rejected	Increasing the share of warnings in all inspections does not lead to a reduction in pollutant air concentration. The tests cannot be considered because strong correlation was observed between the included variables in the model.

Source: Authors’ own calculations and projections.
